# Dimensional schizotypy and social cognition: an fMRI imaging study

**DOI:** 10.3389/fnbeh.2015.00133

**Published:** 2015-05-27

**Authors:** Yi Wang, Wen-hua Liu, Zhi Li, Xin-hua Wei, Xin-qing Jiang, David L. Neumann, David H. K. Shum, Eric F. C. Cheung, Raymond C. K. Chan

**Affiliations:** ^1^Neuropsychology and Applied Cognitive Neuroscience Laboratory, Key Laboratory of Mental Health, Institute of Psychology, Chinese Academy of SciencesBeijing, China; ^2^School of Health Management, Guangzhou Medical UniversityGuangzhou, China; ^3^University of Chinese Academy of SciencesBeijing, China; ^4^Department of Radiology, Guangzhou First People's HospitalGuangzhou, China; ^5^Menzies Health Institute Queensland and School of Applied Psychology, Griffith UniversityGold Coast, QLD, Australia; ^6^Castle Peak HospitalHong Kong, China

**Keywords:** schizotypy, theory of mind, empathy, fMRI, anhedonia

## Abstract

Impairment in empathy has been demonstrated in patients with schizophrenia and individuals with psychosis proneness. In the present study, we examined the neural correlates underlying theory of mind (ToM) and empathy and the relationships between these two social cognitive abilities with schizotypy. Fifty-six first-year college students (31 males, 25 females) between 17 and 21 years of age (*M* = 19.3, *SD* = 0.9) from a medical university in China participated. All participants undertook a comic strips functional imaging task that specifically examined both empathy and ToM. In addition, they completed two self-report scales: the Chapman Psychosis Proneness scale and the Interpersonal Responsivity Index (IRI). Results showed that both empathy and ToM conditions of the task were associated with brain activity in the middle temporal gyrus, the temporo-parietal junction (TPJ), the precuneus and the posterior cingulate gyrus. In addition, we found positive correlations between negative schizotypy and brain activity in regions involved in social cognition, namely, the middle temporal gyrus, the TPJ, as well as the medial prefrontal gyrus. These findings highlight that different dimensions of schizotypy may show different associations with brain regions involved in social cognitive abilities. More importantly, the positive correlation between brain activity and anhedonia suggests the presence of compensatory mechanisms in high-risk populations.

## Introduction

Theory of mind (ToM) refers to the ability to understand others' mental state and infer their aims, intentions, and beliefs (Premack and Woodruff, 1978). It is an important ability that influences social functioning in humans. Deficits in ToM performance in patients with schizophrenia have been well-established (Sprong et al., 2007; Bora et al., 2009). ToM impairment has also been observed in first onset schizophrenia patients, unaffected relatives of schizophrenia patients, ultra-high risk individuals (Bora and Pantelis, 2013), as well as individuals with psychometrically-defined schizotypy (Morrison et al., 2013). However, the mechanism underlying ToM deficit in schizophrenia spectrum disorders and its association with clinical symptoms are not fully understood. Studies that measure dimensions and extent of schizotypy has the advantage of avoiding the confounding effects of medication and illness duration in patients with schizophrenia. Recent studies suggest that schizotypy has multiple dimensions, such as positive schizotypy (e.g., magical ideation and perceptual aberration, similar to positive symptoms in schizophrenia), negative schizotypy (e.g., anhedonia, similar to negative symptoms in schizophrenia) and that these different dimensions may have unique behavioral, cognitive, and brain correlates (Nelson et al., 2013; Ettinger et al., 2014). While some studies found no significant association between schizotypal traits and ToM performance (Jahshan and Sergi, 2007; Fernyhough et al., 2008; McCleery et al., 2012), other studies showed that ToM deficits are related to positive schizotypy (Pickup, 2006; Barragan et al., 2011; Gooding and Pflum, 2011; Pflum et al., 2013) and negative schizotypy (Aldebot Sacks et al., 2012). The multidimensional nature of schizotypy may be an important confounding factor contributing to the inconsistent findings in research on schizotypy.

Concerning the neural correlates of ToM, it has been shown that the medial prefrontal region and the superior temporal sulcus (STS) are involved in ToM processing (Frith and Frith, 2005), and these areas are considered key areas for mentalizing (Blakemore, 2008). In schizophrenia patients, reduction of gray matter volume in the ventral medial prefrontal cortex (Hooker et al., 2011) and the STS (Koelkebeck et al., 2013) are positively correlated with impaired ToM performance. Abnormal activation in the medial prefrontal cortex (MPFC), the STS and the temporo-parietal junction (TPJ) in response to ToM tasks in patients with schizophrenia have also been observed (Brunet-Gouet and Decety, 2006; Bosia et al., 2012). On the other hand, previous studies have found increased brain activation in individuals at-risk of developing schizophrenia (Brune et al., 2011) as well as individuals with psychosis proneness as measured by the positive subscale of the Community Assessment of Psychic Experiences questionnaire (Modinos et al., 2010) to controls. Although preliminary, these results suggest that high risk individuals already show changes in neural activity when performing ToM tasks in the absence of significant behavioral changes.

ToM is now conceptualized as having two components, namely, cognitive, and affective ToM (Shamay-Tsoory et al., 2007): the former refers to making inference about others' beliefs and intentions and the latter refers to making inference about others' emotions. Shamay-Tsoory et al. (2007) suggested that performance on cognitive and affective ToM are dissociated in patients with schizophrenia, and negative symptoms may have unique associations with affective ToM. Empathy is a construct related to ToM and refers to the ability to infer and share others' emotional state, which is an important requirement in social cognition (Green et al., 2008). Empathy has also been considered to have both cognitive and affective components. In terms of psychological processing, affective ToM is similar to the cognitive component of empathy, as both require inferring the emotions of others (Sebastian et al., 2012). In contrast to findings in ToM, empirical findings have shown that negative schizotypy (such as the anhedonia score measured by the Chapman Psychosis Proneness Scale (Chapman et al., 1995) or the “no close friends” subscale score of the Schizotypal Personality Questionnaire (SPQ, Raine, 1991) is associated with empathy (Henry et al., 2008; Wang et al., 2013a).

Vollm et al. (2006) used a functional imaging task that included both ToM and empathy conditions and examined the neural correlates of each condition and their differences in 13 healthy participants (Vollm et al., 2006). The results showed that brain regions like the MPFC, the TPJ and the temporal poles were commonly activated in both conditions, while affective empathy triggered the activation of brain regions involved in emotional processing, such as the cingulate and the amygdala. Adopting a similar paradigm, Benedetti and his colleagues observed abnormal activations in the superior temporal gyrus, the TPJ as well as the prefrontal cortex for both cognitive and affective ToM conditions in chronic schizophrenia patients (Benedetti et al., 2009).

The purpose of the present study was to examine the associations between the various dimensions of schizotypal traits as measured by the Chapman Psychosis Proneness Scale and brain activity during a functional imaging task consisting of both ToM and empathy conditions. We hypothesized that brain areas, including the STS/TPJ and the MPFC, would be activated in both the ToM and empathy conditions of the imaging task. We further hypothesized that positive and negative schizotypy would be associated with different patterns of brain activation under ToM and empathy conditions. Since positive schizotypy have been found to be associated with deficits in ToM performance, and negative schizotypy have been found to be related to mentalization of emotions, we hypothesized that between schizotypal traits and The Interpersonal Reactivity Index (IRI) scores, the association between higher negative schizotypy (anhedonia) and poorer empathic ability would be found. Furthermore, while positive schizotypy may be associated with brain activity changes in the TPJ/STS in the ToM conditions, negative schizotypy may be associated with abnormal brain activity changes in the temporal and parietal lobes in empathy conditions.

## Materials and methods

### Participants

Fifty-six first-year college students (31 males and 25 females) aged between 17 and 21 years (*M* = 19.3 years, *SD* = 0.9) from the Guangzhou Medical University participated in this study. All were right-handed as assessed by the Annett Handedness Scale (Annett, 1970). None had a history of psychiatric disorder, drug abuse or neurological disorders. Participants who scored higher than 13 on the Beck Depression Inventory (Beck et al., 1961) were excluded (four participants). IQ of the participants were estimated using the information, arithmetic, similarity and digit span subtests of the Chinese Version Wechsler Adult Intelligence Scale-Revised (WAIS-R) (Gong and Dai, 1984). The estimated IQ of participants ranged from 88 to 139, with a mean of 118.32 (*SD* = 9.84). The present study was approved by the Ethics Committee of the Institute of Psychology, the Chinese Academy of Sciences. Written informed consent was obtained from each participant.

### Measures

#### Imaging task

A visual ToM functional MRI task consisting of a series of comic strips was used in the present study. It was adapted into Chinese from a task used by Vollm et al. (2006). The task was presented in a block design with eight blocks in total. There were four kinds of stories: ToM, empathy (Emp), physical causality with one character and physical causality with two characters. The ToM and Emp conditions had one or two characters in them. Each kind of story was presented twice and all blocks were presented in a random order. In each block, five trials consisting of comic strips belonging to the same kind of stories were presented. In each trial, three pictures depicting a short story were displayed in the upper half of the screen for 6 s. Next, two pictures appeared in the lower half of the screen for another 6 s. During the second 6-s period, participants were asked to choose one of the two pictures from the lower half of the screen as the appropriate ending to the story by pressing the corresponding button. One trial lasted for 12 s; and each block consisted of an initial instruction slide and five trials and lasted for 66 s in total. The duration of the whole task was 8 min and 48 s. For a more detailed description of this task, please refer to Vollm et al. (2006) and Neumann et al. (2014). Before entering the scanner, all participants were given time to practice to make sure that they understood the task.

#### Self-report scales

All participants were also asked to complete self-report scales that measure schizotypal traits and empathy. *The Chinese version of the Chapman Psychosis-Proneness Scales* (Chapman et al., 1995), including the Revised Social Anhedonia Scale (40 items), the Physical Anhedonia scale (61 items), the Magical Ideation Scale (40 items), and the Perceptual Aberration Scale (35 items) were used to determine the positive and negative dimensions of schizotypy (Wang et al., 2012). The former two Chapman scales were used to capture negative schizotypal traits (anhedonia) and the latter two scales were used to capture positive schizotypal traits (psychotic-like positive symptoms). The higher the participants scored, the higher was their level of schizotypy. The Cronbach's coefficients alpha of the four scales ranged from 0.75 to 0.89 in the present study.

*The Interpersonal Reactivity Index* (IRI) is a 28-item self-report scale that measures empathy and it consists of four subscales: perspective taking, fantasy, personal distress, and empathic concern (Davis, 1983). The first two subscales were designed to capture cognitive empathy and the last two scales captured affective empathy. In the Chinese version of the IRI, six items were deleted and the remaining 22 items have been shown to have good reliability and validity in both normal and schizophrenia populations (Chan, 1986). The Cronbach's alpha coefficients were 0.82 for the personal distress, 0.77 for the perspective taking, 0.73 for the fantasy and 0.63 for the empathic concern subscale.

### Images acquisition and preprocessing

All MRI scans were acquired using a 3T SIEMENS Verio MR scanner at the Guangzhou First People's Hospital, Guangzhou, China. Functional imaging data were acquired using a T2-weighted echo planar imaging (EPI) sequence; 264 whole-brain volumes were collected with slice thickness = 4 mm, echo time (TE) = 28 ms, repetition time (TR) = 2000 ms, flip angle = 90°, matrix size = 64 × 64, 32 slices in coronal plane, field of view (FOV) = 210 × 210 mm, voxel size = 3 × 3 × 4 mm, bandwidth = 2232 Hz/Px. Scans were screened by a radiologist to exclude any incidental clinical abnormalities before further analyses. Preprocessing was performed using the Statistical Parametric Mapping software (SPM8, Wellcome Department of Imaging Neuroscience, Institute of Neurology and the National Hospital for Neurology and Neurosurgery; London, England). The first eight volumes were removed. Time delay in image acquisition and head motion were corrected. The fMRI images were further spatially normalized to the Montreal Neurological Institute (MNI) EPI template and re-sliced to 3 mm cubic voxels and then smoothed using a 10 mm full width at half maximum (FWHM) Gaussian kernel. Using the general linear model, contrast images, and beta images were generated for each participant, including “ToM minus physical causality with one character,” and “Empathy minus physical causality with two characters.” The head motion parameters in six directions were taken as covariates.

### Statistical analysis

For the behavioral data, we calculated the descriptive statistics of the scores on the self-report scales, including the IRI and the Chapman scales, as well as performance on the imaging task. Since gender has been identified as a confounding variable in previous empathy studies (e.g., Wang et al., 2013a), we also examined gender differences for the behavioral variables. To examine the associations between schizotypy and ToM/empathy, we calculated correlations between dimensional schizotypal traits measured by self-report scales and the IRI scores.

For the functional imaging data, all participants' contrast images, including contrast images for the ToM condition modeled as “ToM minus physical causality with one character” and the empathy condition modeled as “Empathy minus physical causality with two characters” were taken into the second level analysis. We first conducted one-sample *t*-tests using the contrast images for ToM and empathy conditions to examine brain activation in both conditions. In addition, we also conducted conjunction analysis to confirm the common areas for both conditions. The clusters were reported with a threshold of *p* < 0.001 and a cluster size of >50 voxels (corresponding to a cluster-level AlphaSim corrected *p* < 0.01 for multiple comparisons). After identifying the brain regions involved in the ToM and/or empathy conditions, we extracted the percentage signal change of those regions during the ToM and empathy blocks and calculated the correlations between the percentage signal change and schizotypy scores on the Chapman scales. Regions of interest were defined according to the results of the one sample *t*-tests and conjunction analysis. The percentage signal changes of all ROIs for each participant were extracted using the Marsbar v0.43 (Matthew et al., 2002) toolbox.

## Results

### Self-reported scores and behavioral performance

Table 1 shows age, estimated IQ, IRI scores and the Chapman Psychosis Proneness scores for the whole sample. Results of independent sample *t*-tests showed that female participants reported higher scores on the IRI personal distress subscale (*t* = 4.20, *p* < 0.001). Concerning the behavioral performance in the imaging task, the mean accuracy across all trials were higher than 0.80, and there was no significant gender effect except that males had higher accuracy in the empathy condition (*t* = 2.61, *p* < 0.05).

**Table 1:** **Descriptive analyses and gender effect on behavioral results**.

	**Whole sample (*n* = 56)**		**Male (*n* = 31)**		**Female (*n* = 25)**		**Gender effect**	
	**Mean**	**SD**	**Mean**	**SD**	**Mean**	**SD**	***t***	***p***
Age	19.25	0.88	19.35	0.95	19.12	0.78	1.02	0.315
IQ estimates	118.32	9.84	117.42	9.56	119.44	10.26	−0.76	0.450
**IRI SCALE**								
PD	2.65	0.72	2.33	0.61	3.04	0.65	−**4.20**	**0.000**
PT	3.78	0.51	3.81	0.58	3.74	0.42	0.56	0.582
FA	3.56	0.67	3.42	0.70	3.73	0.60	−1.79	0.079
EC	3.71	0.54	3.63	0.51	3.81	0.57	−1.28	0.208
**SCHIZOTYPY**								
CSAS	7.80	5.58	8.16	6.26	7.36	4.68	0.53	0.598
CPAS	12.36	10.35	12.87	10.95	11.72	9.75	0.41	0.683
PAS	6.87	7.54	7.32	9.02	6.32	5.28	0.49	0.625
MIS	10.73	5.75	9.68	5.02	12.04	6.41	−1.55	0.128
**IMAGING TASK (ACCURACY)**								
ToM	0.80	0.16	0.83	0.14	0.76	0.18	1.62	0.112
Empathy	0.94	0.08	0.96	0.07	0.91	0.09	**2.61**	**0.012**
Physical causality with one character	0.90	0.11	0.92	0.10	0.88	0.12	1.23	0.223
Physical causality with two characters	0.83	0.15	0.86	0.15	0.80	0.15	1.36	0.180

*Two sample t-tests were conducted to explore the gender effect, two-tailed. IRI, The Interpersonal Reactivity Index; PD, personal distress; PT, perspective taking; FA, fantasy; EC, empathic concern; CSAS, Chinese version of social anhedonia scale; CPAS, Chinese version of physical anhedonia scale; PAS, Chinese version of perceptual aberration scale; MIS, Chinese version of magical ideation scale; ToM, Theory of Mind. Values in bold indicate that they are statistical significant*.

### Functional imaging results

**Figure 1:**
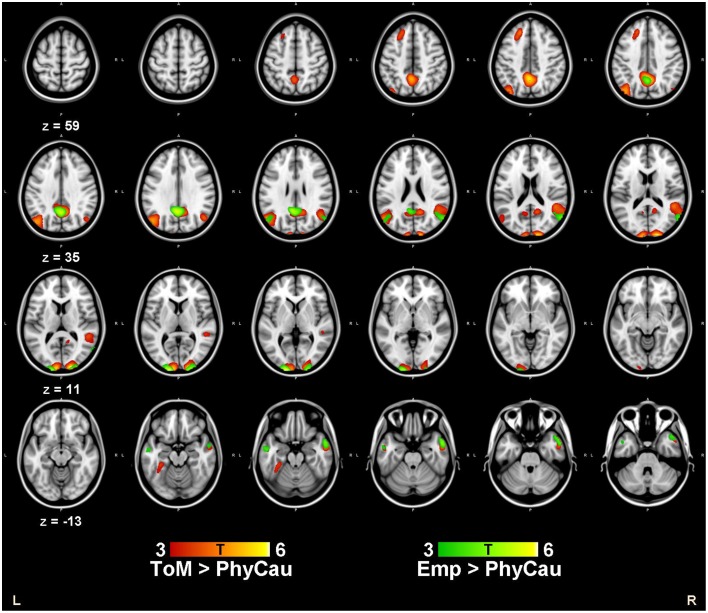
**The Brain activations associated with the ToM and Empathy conditions**. Compared to the control condition (physical causality with one character), increased brain activity were found in bilateral cuneus, bilateral middle temporal gyrus, left precuneus, left superior frontal cortex and the right TPJ during the ToM blocks. For the empathy condition, increased brain activations were found in bilateral cuneus, left precuneus and also the right middle temporal gyrus. One sample *t*-tests were conducted for ToM and Empathy conditions, respectively. Threshold was set at *p* < 0.001, cluster size >50 voxels.

We conducted a series of one sample *t*-tests to examine the brain activation associated with the ToM and empathy conditions. Compared to the physical causality condition, we found increased activity in the bilateral cuneus, the bilateral middle temporal gyrus, the left precuneus, the left superior frontal cortex and the right TPJ during the ToM condition. For the empathy condition relative to the physical causality condition, we found a similar increase in brain activations in the bilateral cuneus, the left precuneus and the right middle temporal gyrus. The results are shown in Table 2 and Figure 1. Using conjunction analysis, we further confirmed the common areas activated by both the ToM and the empathy conditions, namely the bilateral cuneus, the middle temporal gyrus and the TPJ (Table 2). Further comparisons showed that the left TPJ and the lingual gyrus were activated more strongly during the ToM condition than the empathy condition.

**Table 2:** **Brain activations associated with ToM and Empathy conditions**.

	**All participants (*n* = 56)**					
	**BA**	**Coordinate**			**Cluster**	**Peak**
		***x***	***y***	***z***	**size**	**(Z)**
**TOM MINUS PHYSICAL CAUSALITY**						
Cuneus	BA17/18	18	−102	12	207	5.64
Precuneus/posterior cingulate	BA7/31	−3	−54	42	466	5.05
Cuneus	BA17	−9	−102	9	209	4.89
Middle temporal/precuneus	BA 19/39	−39	−78	42	285	4.59
Middle temporal gyrus	BA21	54	0	−27	106	4.32
Superior frontal gyrus, extending to cingulate	BA8/32	−18	33	42	75	4.19
Temporal-parietal junction	BA 13/22/39	48	−45	15	300	4.06
**EMPATHY MINUS PHYSICAL CAUSALITY**						
Cuneus/lingual gyrus	BA 18	−18	−105	3	84	5.48
Precuneus/posterior cingulate	BA7/31	0	−57	33	183	5.14
Cuneus	BA 18	21	−105	6	50	4.66
Middle temporal gyrus	BA 21/38	54	6	−27	90	4.25
Middle temporal gyrus	BA39	60	−66	18	67	3.89
**CONJUNCTION ANALYSIS**						
Cuneus	BA17/18	18	−105	9	99	6.86
Precuneus/posterior cingulate	BA7/31	0	−57	36	251	6.65
Cuneus	BA17/18	−15	−105	6	106	6.51
Middle temporal gyrus	BA 21/38	54	6	−27	100	5.95
Temporal-parietal junction	BA 39	−51	−72	27	107	5.53
Temporal-parietal junction	BA 39	54	−66	21	105	5.21
Middle temporal gyrus	BA 21/38	−54	−6	−21	22	5.05
**(TOM—PHYSICAL CAUSALITY) MINUS (EMPATHY—PHYSICAL CAUSALITY)**						
Temporal-parietal junction	BA 39	−42	−72	39	94	4.10
Cuneus/lingual gyrus	BA17/18	−3	−93	6	253	3.89
**(EMPATHY—PHYSICAL CAUSALITY) MINUS (TOM—PHYSICAL CAUSALITY)**						
No significant cluster						

*One sample t-tests were conducted for Theory of Mind (ToM) and Empathy conditions, respectively. Threshold was set at p < 0.001, cluster size > 50 voxels. For the conjunction analysis, the threshold was set at p < 0.05, family-wise error correction. ToM, Theory of Mind; BA, Brodmann Area*.

### Associations between schizotypy and brain activity

We then extracted the percentage signal changes of significant clusters related to the ToM or the empathy conditions in our imaging task. We calculated the correlations between schizotypy and IRI scores, as well as the percentage signal changes of ROIs, with and without gender as covariates.

We found significant negative correlations between social anhedonia and IRI empathic concern subscale scores (*r* = −0.24, *p* < 0.05), between physical anhedonia and IRI fantasy subscale scores (*r* = −0.30, *p* < 0.05) and IRI empathic concern subscale scores (*r* = −0.35, *p* < 0.01). We also found positive correlations between magical ideation subscale scores and IRI personal distress subscale scores (*r* = 0.30, *p* < 0.05) and fantasy subscale scores (*r* = 0.30, *p* < 0.05). Using gender as covariate, we also found significant positive correlations between both physical and social anhedonia and IRI personal distress subscale scores (see Table 3 for details).

**Table 3:** **The correlations between schizotypy and IRI, percent signal change**.

	**Correlation without covariate**				**Correlation with gender as covariate**			
	**CSAS**	**CPAS**	**PAS**	**MIS**	**CSAS**	**CPAS**	**PAS**	**MIS**
**IRI SCALE**								
PD	0.19 (0.085)	0.18 (0.090)	0.06	0.30^*^	0.26^*^	0.24^*^	0.11	0.23^*^
PT	−0.06	0.02	0.02	0.01	−0.06	0.01	0.01	0.03
FA	−0.18 (0.097)	−0.30^*^	−0.04	0.30^*^	−0.16	−0.30^*^	−0.03	0.26^*^
EC	−0.24^*^	−0.34^**^	−0.06	0.09	−0.23^*^	−0.34^**^	−0.05	0.06
**% SIGNAL CHANGE OF ROIS (CONTRAST, COORDINATES X, Y, Z)**								
Cuneus (ToM, 18, −102, 12)	0.20 (0.075)	0.06	0.09	−0.05	0.18 (0.090)	0.05	0.08	0.00
Temporal-parietal junction (ToM, 48, −45, 15)	0.27^*^	0.09	0.14	0.04	0.27^*^	0.09	0.14	0.04
Middle temporal gyrus (ToM, 54, 0, −27)	0.23^*^	0.05	0.13	0.04	0.23^*^	0.05	0.13	0.04
Precuneus (ToM, −3, −54, 42)	0.15	0.13	0.06	−0.09	0.15	0.13	0.05	−0.07
Cuneus (ToM, −9, −102, 9)	−0.01	−0.03	0.02	−0.04	−0.02	−0.04	0.00	0.00
Superior frontal gyrus (ToM, −18, 33, 42)	0.21 (0.063)	0.09	−0.03	−0.22 (0.051)	0.20 (0.068)	0.09	−0.03	−0.21 (0.058)
Middle temporal gyrus (ToM, −39, −78, 42)	0.24^*^	0.23^*^	0.05	−0.11	0.24^*^	0.23^*^	0.05	−0.11
Precuneus (EMP, 0, −57, 33)	0.09	0.01	0.13	−0.05	0.09	0.02	0.14	−0.07
Cuneus (EMP, 21, −105, 6)	0.23^*^	0.13	0.01	0.02	0.23^*^	0.13	0.00	0.04
Middle temporal gyrus (EMP, 54, 6, −27)	0.14	0.07	0.15	0.11	0.17	0.09	0.18 (0.097)	0.06
Middle temporal gyrus (EMP, 60, −66, 18)	0.22^*^	−0.05	0.13	0.08	0.22 (0.051)	−0.05	0.13	0.08
Cuneus (EMP, −18, −105, 3)	−0.06	−0.19 (0.083)	0.14	0.21 (0.061)	−0.06	−0.18 (0.089)	0.15	0.20 (0.070)

*Correlation and partial correlations with gender as covariate were conducted, threshold was set as p < 0.05 one-tailed. IRI, The Interpersonal Reactivity Index; PD, personal distress; PT, perspective taking; FA, fantasy; EC, empathic concern. CSAS, Chinese version of social anhedonia scale; CPAS, Chinese version of physical anhedonia scale; PAS, Chinese version of perceptual aberration scale; MIS, Chinese version of magical ideation scale; ToM, Theory of Mind; EMP, Empathy*.

* p < 0.05;

** *p < 0.01*.

*The p-values are presented in parentheses were between 0.05 and 0.10*.

*Values in bold indicate that they are statistical significant*.

Concerning the correlations with percentage signal changes, in the ToM condition, positive correlations were found between social anhedonia and brain activity in the right cuneus, the bilateral middle temporal gyrus, the medial frontal gyrus, and the right TPJ. We also found significant positive correlations between physical anhedonia scores and brain activity in the left middle temporal gyrus. Marginally significant correlations were also found between magical ideation subscale scores and activation in the medial frontal gyrus. For the empathy condition, positive correlations were found between social anhedonia scores and brain activity in the right cuneus and the middle temporal gyrus. The correlation between the MIS scores and brain activity in the right cuneus reached trend significance (see Table 3).

## Discussion

In the present study, we adopted a comic strips functional imaging task to explore the brain regions involved in the mentalizing process of ToM and empathy. We found several brain regions related to the process of inferring others' beliefs, including the TPJ, the middle temporal gyrus, the medial prefrontal cortex, the cuneus and the precuneus. We found that similar brain regions, namely, the cuneus, the precuneus and the middle temporal gyrus, were also activated when inferring the emotional state of others. By using conjunction analysis, we found regions such as the bilateral TPJ, the middle temporal gyrus and the cuneus to be activated in both conditions. In addition, we explored the associations between schizotypal traits and brain activities in these clusters. The results showed that social anhedonia scores, a negative dimension of schizotypy, had negative correlations with self-report empathy, but they were positively correlated with brain activity of the cuneus, the middle temporal gyrus and the TPJ, which are brain regions involved in ToM/empathy. For positive schizotypal traits, scores on the magical ideation subscale of the Chapman scales were negatively correlated with brain activity of the medial prefrontal cortex.

Key brain regions involved in the attribution of mental states, the so-called “social brain,” include the medial prefrontal gyrus, the posterior STS/TPJ, the middle temporal gyrus, the anterior cingulate and the anterior insula (Blakemore, 2008). Adopting the comic strips task, Vollm et al. (2006) found that the medial prefrontal gyrus, the TPJ and the temporal pole were activated in both ToM and empathy processing tasks and suggested that both processes rely on the brain network associated with making inferences of others' mental states. The posterior STS/TPJ integrates information from the external environment and internal sensory and perceptual information and plays an important role in self-other distinction (Abu-Akel and Shamay-Tsoory, 2011). Others have also suggested that the TPJ is selectively activated in the attribution of mental states and plays a specific and independent role in the prediction of the behavior of others (Saxe and Wexler, 2005; Carter et al., 2012). In our study, both the ToM and empathy tasks required participants to infer the intentions of the character in the comic strips and choose the most appropriate ending of the story. As expected, we found increased brain activation in the bilateral TPJ (BA39) and the middle temporal gyrus (BA21/38) during the ToM and empathy conditions compared to the physical causality blocks. At the same time, we also found increased activation in the middle occipital gyrus (cuneus, BA18) in the conjunction analysis, which has also been reported previously (Vollm et al., 2006).

Increased activation in the posteromedial regions, including the posterior cingulate and the precuneus, was consistently found under both ToM and empathy conditions in our study. These findings are consistent with previous studies on mentalizing tasks (Vollm et al., 2006; Abu-Akel and Shamay-Tsoory, 2011). The precuneus is involved in self-processing, mental imaginary as well as episodic memory retrieval (Cavanna and Trimble, 2006) and all these processes might be related to inferring the intention of others. For example, during the mentalizing task, participants needed to imagine the scene or story of the characters in the comic strip and infer their emotional states. Abu-Akel and Shamay-Tsoory (2011) have proposed a model to explain the neuroanatomical basis of ToM, in which the posterior cingulate and the precuneus are involved in representing and distinguishing self from the mental states of others.

Compared to the empathy condition, increased activation in the temporal-parietal junction and the cuneus were found during the ToM condition. This difference might be related to the difference in difficulty of the ToM and empathy conditions. In the present study, the mean accuracy of the ToM and empathy condition was 0.80 and 0.94, respectively. The difference in accuracy might be a reflection of the difficulty of the ToM condition.

Consistent with previous findings, we found that a higher level of negative schizotypy was correlated with lower scores on the IRI scale (Henry et al., 2008; Wang et al., 2013a). With or without gender as covariate, we found significant correlations between negative schizotypy (social or physical anhedonia) and IRI fantasy/empathic concern scores, suggesting that individuals with higher social or physical anhedonia scores had poorer self-report empathic ability. As components of social cognition, ToM and empathy play important roles in social functioning (Schmidt et al., 2011). Our results suggest that schizotypy, especially the negative dimension of schizotypy, is associated with poorer social cognition, which may explain the deficits in social functioning in individuals with high-risk of developing schizophrenia and individuals with schizotypal traits, which is consistent with previous studies (Addington et al., 2011; Blanchard et al., 2011; Wang et al., 2013b).

Most interestingly, we extracted the percentage signal change of the activated brain regions and calculated the correlations with both positive and negative schizotypy traits. We found that social anhedonia was positively correlated with brain activity in several regions involved in ToM/empathy processing, including the bilateral middle temporal gyrus, the medial frontal gyrus and the right TPJ. In a previous study that adopted a similar task, Benedetti et al. (2009) found that patients with schizophrenia showed higher brain activation in their temporal and frontal lobes. Although reduced brain activities has been found in the frontal, temporal and parietal lobes of patients with schizophrenia in different functional imaging studies, an inverse pattern of brain activation has also been reported in high-risk populations. For example, increased activity was found in a visual imaging ToM task in individuals with high psychosis proneness measured by the Community Assessment of Psychic Experiences Questionnaire (CAPE) (Modinos et al., 2010). Using resting state fMRI techniques, researchers have also found positive correlations between SPQ scores and visual network in adolescents (Lagioia et al., 2010). Taken together, we believe that the increased brain activation observed in high risk populations may represent some form of compensatory or protective mechanism, which could be a valuable target for future studies in psychosis development.

We acknowledge several limitations in the present study. First, for the behavioral tasks that captured mental state attribution, we only used a self-report scale as there is a lack of appropriate behavioral paradigms suitable for young adults with good validity. Secondly, for the imaging data analysis, no stringent correction for multiple comparisons was used. Nevertheless, it should be pointed out that the threshold was set at *p* < 0.001 and cluster size was more than 50 voxels, which corresponded to a cluster level of *p* < 0.01 (AlphaSim correction for multiple comparison). It should also be noted that we did not adopt multiple comparison adjustment for the correlation analyses between the percentage signal change and schizotypy scores on the Chapman scales, and so the results would need to be considered cautiously. Thirdly, our findings were limited to healthy individuals with schizotypal traits. Finally, the comic strips task does not isolate the individual subcomponents of ToM and empathy. For example, empathy is considered to consist of components such as affect sharing, perspective taking, and understanding others' situation (Neumann et al., 2013). Further research that uses a modified version of the comics strips task to examine the relationships between schizotypy and brain activation under different empathy conditions is needed.

In conclusion, our results led us to postulate that negative schizotypy may play an important role in social cognition processing, such as mentalizing, which may further influence an individual's social functioning. The inverse correlation pattern between brain activity and positive and negative schizotypy strengthens the idea that the multidimensional structure of schizotypy is complex and should be examined more systematically in future studies.

### Conflict of interest statement

The authors declare that the research was conducted in the absence of any commercial or financial relationships that could be construed as a potential conflict of interest.
